# Supplementary material for: In vitro activity of AST-120 that suppresses indole signaling in Escherichia coli, which attenuates drug tolerance and virulence
Source: PLoS One. 2020 Apr 29;15(4):e0232461. doi: 10.1371/journal.pone.0232461 (PMC7190153; doi:10.1371/journal.pone.0232461)
Supplement: Attachment — Submitted filename: Response to Reviewers.docx [file pone.0232461.s001.docx]

Editor’s comment: Thank you for submitting your manuscript to PLOS ONE. After careful consideration, we feel that it has merit but does not fully meet PLOS ONE’s publication criteria as it currently stands. Therefore, we invite you to submit a revised version of the manuscript that addresses the points raised during the review process.
The present methodology does not support the results and authors' conclusions. The authors should perform substantial changes to the methodology so as to address reviewers comments.

**Response: As suggested, we revised our manuscript, and particularly improved the methodology. We believe that the revised manuscript now satisfies both reviewers. Our responses to reviewer’s comments are described as below.**

Reviewer #1: This manuscript titled“In vitro Activity of AST-120 That Suppresses Indole Signaling in Escherichia coli, Which Attenuates Drug Tolerance and Virulence” describes a series of conclusions, and the experimental results are very detailed. However, the current status cannot be published yet, and the following modifications need to be completed before publication.

1. The introduction of indoles is not comprehensive enough, and there is a lack of multiple references, such as the recent studies by Thomas K Wood. Several new developments regarding Lysobacter were also not mentioned. These need to be added.

**Response: We think that it may be better to add some of references into Discussion in addition to Introduction. We added some of works published by Thomas K. Wood into Introduction and Discussion as references, and a Lysobacter work into Discussion (Please see Lines 73-74 and Lines 417-422 in the marked-up copy file).**

2. In Figure1, the significant change in statistics is only shown as P<0.01 and is only represented by single asterisk. The author needs to use one, two, and three asterisks for different P values.

**Response: As suggested, we did it (Please see Fig 1A, Fig 1B Fig 2A, Fig 2B and Fig 4A).**

3. In figure 1, the effect of 10mg ast-120 is obvious, what about 1mg or 5mg? This data needs to be shown in the diagram. This series of data needs to be improved.

**Response: We tested the effect of 1mg, 2mg and 5mg AST-120, and presented in the diagram figures (as Fig1A and Fig1B). Fig 1C in the original version was renamed as Fig 1B because Fig1B in the original version was removed as suggested by reviewer 2.**

4. English writing needs polishing.

**Response: Again, we carefully checked English writing.**

5. There are many studies on the regulation of drug resistance by indoles. Previous studies have shown that indoles increase drug resistance in some bacteria and inhibit drug resistance in others. Are these background studies relevant to this study? Is there any inspiration? Need to be reflected in the discussion.

**Response: Thank you for your suggestion. In contrast to *E. coli*, intrinsic antibiotic resistance (or tolerance?) of *Lysobacter* is reduced by indole (Wang et al., mBio, 2019). As other cases, indole attenuates virulence of *P. aeruginosa* and Staphylococcus aureus (Lee et al., Microb. Biotechnol, 2009)(Lee et al., Appl. Microbiol. Biotechnol., 2013) and also inhibits *P. aeruginosa* persister cell resuscitation (Zhang et al., J. Appl. Microbiol, 2019). Therefore, we have to keep in our mind that AST-120 may exhibit detrimental aspects in some of cases for other bacterial species. However, we believe that AST-120 still has a merit for treatment of *E. coli* infections including EHEC. We added this statement into the discussion with adding these references (Please see Lines 417-422 in the marked-up copy file).**

Reviewer #2: The manuscript describes a novel observation that AST-120 (Kremezin), used clinically for chronic kidney disease, is capable of adsorbing indole (in addition to a range of other molecules). One would naturally conclude that the addition of AST-120 to a bacterial culture will interfere with most indole-related signalling pathways, not because AST-120 is interfering in these pathways but simply because indole is absent/decreased. The authors demonstrate this expected outcome in a range of assays (antibiotic tolerance, regulation of genes encoding efflux pumps and fosfomycin permease & secretion of EspB protein). However, the authors claim that AST-120 interferes in these pathways. This claim is unsubstantiated as AST-120 has no effect on these pathways if indole is not present, as shown by their results. Therefore, these assays simply confirm the initial observation of AST-120 adsorbing indole without providing any added significance.

Abstract:
Lines 40-42: The results suggest only that AST-120 can adsorb a range of compounds. There is no data to support that a combination therapy of AST-120 with quinolones or B-lactams will lead to a better clinical outcome, hence the statement is unjustified.

**Response: We deleted this sentence.**

Introduction:
Lines 73: The authors should explain the difference between resistance and tolerance for the benefit of the lay audience, I suggest referring to Nature Reviews Microbiology 14, 320 (2016) & mBio 10, e02095-19 (2019). Tolerant and persistent bacteria can survive antibiotic treatments without having acquired a resistance phenotype. In contrast to resistance, which enables bacteria to grow in a concentration of a drug that would otherwise prevent growth, tolerance is only a transient ability of bacteria to survive under otherwise bactericidal treatments.

**Response: As suggested, we added the literature of resistance and tolerance together with suggested references (Please see Lines 53-59 in the marked-up copy file).**

Lines 75-81: The authors describe indole as a detrimental molecule on the host. This is inaccurate as indole has been reported to have both positive and negative effects (For example, see FEMS Nicrobiol Rev 34, 426-44 (2010) & Trends Microbiol 23, 707-718 (2015)).

**Response: We were aware that indole also has positive roles, and we have described in the discussion. In this section, we just mention that indole causes accumulation of indoxyl-sulfate, a uremic toxin. To avoid confusion we removed the sentence of “From the host’s standpoint, indole is a waste metabolite” (Described in Line75 in original version).**

Materials and Methods:
Lines 123-131: “Drug susceptibility assays” is described vaguely (what is the starting cfu/ml before treatment? Why incubation with no shaking? Why 60 min incubation for the antibiotics but 30 min for rhodamine G?). Also, the choice of the technique is odd.
As a starting point, MICs should be assayed for all tested antibiotics (and Rhodamine G) before and after the addition of AST-120 to confirm that we are looking at an increased tolerance not increased resistance. In contrast with resistance, tolerance do not lead to an increase in the MIC compared with susceptible bacteria (See Nature Reviews Microbiology 17, 441-448 (2019)). Next, tolerance is characterised by a slower killing even at high concentrations of the drug, and that is assayed by either MDK99 (the minimum duration of treatment that kills 99% of the bacterial population) (See Nat Rev Microbiol 14:320–330 (2016)) or TDtest (see Sci Rep 7, 41284 (2017). The authors need to comment on the suitability of their chosen method, considering that it is not how tolerance is usually assayed, unless the authors do not mean actually “tolerance”!

**Response: Thank you for important suggestions. Originally, our understanding was that tolerance is a transient decrease in drug susceptibility without any genetic modifications. We now definitely agree with the reviewer’s suggestion. We carefully checked our statements to differentiate tolerance and resistance through the text, and modified them. As suggested, we initially performed MIC assays for strains grown with and without indole in medium containing aztreonam, fosfomycin and rhodamine 6G, however there was no difference in MICs. Following MIC assays, we measured survival rates in multiple time points, and found that indole-treated cells were slowly killed by those drugs compared to cells grown without indole. We also tested the effect of AST-120, and found that cells grown in the presence of AST-120 with indole were killed as cells grown without indole. Therefore, we believe that indole increases tolerance, and AST-120 abolishes the effect of indole on increase of tolerance. We have now replaced Fig 3, and also modified the text, accordingly (Please see Lines 136-151, 253-275 in the marked-up copy file). In addition, we accordingly added the Nature Reviews Microbiology paper as a reference.**

Lines 146-147: What is the basis of using OD280nm to calculate the concentrations of antibiotics? Why is it changes to 525nm for Rhodamine G?

**Response: The reason why we used 525 nm wavelength to measure rhodamine 6G is that its maximum absorption wavelength is 525nm, therefore we used 525 nm because of high sensitivity for detection. Of course, rhodamine 6G can absorb 280 nm due to the structure containing an aromatic ring. To simply our experiment, we used 280 nm to calculate the concentration of rhodamine 6G as well as other compounds. We replaced the data in Fig 5.**

Lines 148-149: The disk diffusion assays will be influenced by the presence of AST-120. I am wondering if the authors have tested whether AST-120 alone has any inhibitory effect on MG1655?

**Response: The disk diffusion assays was performed to calculate concentrations of fosfomycin, colistin and amikacin in supernatant after removing AST-120. Therefore, AST-120 is not contained in MG1655 culture plate for disk assays.**

Results:
Line 170: What does “authentic” indole means?

**Response: We deleted “authentic”.**

Lines 172-175: The authors justify using 2 mM indole by describing that cultures of stationary phase E. coli in LB contain around 2 mM indole. This is buzzling as the concentration is known to be in the range of 0.3 – 0.7 mM, which is nowhere near 2 mM. See for example
- Di Martino, P., et al., Indole can act as extracellular signal to regulate biofilm formation of Escherichia coli and other indole-producing bacteria. Canadian journal of Microbilogy, 2003. 49(7): p. 443-449.
- Domka, J., J. Lee, and T.K. Wood, YliH (BssR) and YceP (BssS) Regulate Escherichia coli K-12 Biofilm Formation by Influencing Cell Signalling. Appl. Environ. Microbial., 2006. 72(4): p. 2449-2459.
- Hirakawa, H., et al., Secreted indole serves as a signal for expression of type III secretion system translators in enterohaemorrhagic Escherichia coli O157 : H7. Microbiology, 2009. 155(2): p. 541-550.
The authors need to comment on this contradiction.

**Response: Indole production depends on tryptophan. As far as we know, when *E. coli* is cultured in LB, indole concentration in supernatant would be ~0.5 mM. As shown in this experiment, when tryptophan is added into *E. coli* LB culture, the indole concentration in supernatant reaches ~2mM. We added our comment that exogenous tryptophan increases indole production of *E. coli* (Please see Lines 205-208 in the marked-up copy file).**

Lines 177-178 & Fig 1B: The authors have shown than AST-120 adsorbs tryptophan as well as indole. Thus, when adding AST-120 to a tryptophan containing media, it is not possible to determine whether the reduction of indole is due to AST-120 adsorption of indole or tryptophan? This experiment is rather messy with no clear conclusion.

**Response: We removed Fig 1B, now Fig 1C in original version were renamed as Fig 1B in revised manuscript.**

Lines 214-215: The authors conclude that “AST-120 suppresses AcrD-, MdtEF-, and GlpT-associated drug tolerance induced by indole”. This is not what the results show. The results simply show that AST-120 adsorbs indole (almost completely). This means that indole effects will be abolished by the addition of AST-120. Not because AST-120 interferes in the indole effects but simply because indole is not there anymore. In other words, AST-120 does NOT suppress AcrD-, MdtEF-, and GlpT-associated drug tolerance, but simply adsorbs indole so these effects are not there anymore.
Lines 216-222 & Fig 2B: Similar to my previous point, the addition of AST-120 did not affect the level of LacZ expression directly but simply adsorbed indole, thereby the expression of LacZ was decreased.

**Response: We agree that AST-120 does not suppress drug tolerance with promoter activities, but it absorbs indole, and abolishes the indole effects. We modified the text, accordingly (Please see Lines 295-296 in the marked-up copy file).**

Lines 232-233: (Fig. 3A & B) should be (Fig. 4A & B).

**Response: We changed, thank you for catching.**

Lines 237-238: Again, AST-120 does NOT decrease EspB levels as claimed by the authors. AST-120 adsorbs indole thus the WT strains behaves exactly as a ΔtnaA strain.

**Response: Similarly, we modified the text (Please see Lines 347-348 in the marked-up copy file).**
